# Towards Scarless Wound Healing: A Comparison of Protein Expression between Human, Adult and Foetal Fibroblasts

**DOI:** 10.1155/2014/676493

**Published:** 2014-01-30

**Authors:** Sonia Ho, Helder Marçal, Leslie John Ray Foster

**Affiliations:** Bio/Polymer Research Group, School of Biotechnology & Biomolecular Sciences, University of New South Wales, Sydney, NSW 2052, Australia

## Abstract

Proteins from human adult and foetal fibroblast cell lines were compared, focusing on those involved in wound healing. Proteins were separated through two-dimensional gel electrophoresis (2DE). Differences in protein spot intensity between the lineages were quantified through 3D gel scanning densitometry. Selected protein spots were excised, subjected to tryptic digests, prior to separation using HPLC with a linear ion trap mass spectrometer, and identified. Protein maps representing the proteomes from adult and foetal fibroblasts showed similar distributions but revealed differences in expression levels. Heat shock cognate 71 kDA protein, Tubulin alpha-1A chain, actin cytoplasmic-1, and neuron cytoplasmic protein were all expressed in significantly higher concentrations by foetal fibroblasts, nearly double those observed for their adult counterparts. Fructose bisphosphate aldolase A, Cofilin-1, Peroxiredoxin-1, Lactotransferrin Galectin-1, Profilin-1, and Calreticulin were expressed at comparatively higher concentrations by the adult fibroblasts. Significant differences in the expression levels of some proteins in human adult and foetal fibroblasts correlated with known differences in wound healing behaviour. This data may assist in the development of technologies to promote scarless wound healing and better functional tissue repair and regeneration.

## 1. Introduction

Approximately 55 million elective and 25 million trauma operations are performed per annum in the developed world, a total of 80 million patients that acquire various degrees of scar tissue in their dermis [1]. Thermal injury is the most common cause of acute scarring, with trauma, the removal of extensive skin cancer, and medical conditions such as deep fungal and bacterial infections, autoimmune diseases, and vascular complications as other causes of significant acute cutaneous wounds [2–4]. The effects of scarring may be physical, aesthetic, and psychological, with significant health, social, and financial consequences.

The skin is the largest organ in the mammalian body and consists of two major layers, separated by a basement membrane [5]. The waterproof outer epidermis layer serves as a barrier to infection while the underlying dermis, a thick layer of collagen-rich connective tissue, is where appendages unique to the skin are embedded [6]. Upon wounding, the inflammatory phase of normal healing proceeds with endothelial cells, keratinocytes, and fibroblasts undergoing changes in gene expression and phenotype, due to the expression of cytokines such as platelet and fibroblast-derived growth factors (PDGF and FGF) [7]. These changes subsequently lead to cell proliferation, differentiation, and migration [6]. Noncellular components, such as fibrin and collagen, and cellular components of the immune system as well as the blood coagulation cascade and inflammatory pathways are also activated, resulting in a temporary repair achieved in the form of a clot that seals the defect [8]. Angiogenesis, granulation tissue and extracellular matrix (ECM) formation, and reepithelisation subsequently take place during the proliferation phase, characterized by cellular proliferation and migration to the wound site [7, 9]. The final remodelling phase begins 2 to 3 weeks after injury and often takes weeks, months, or even years to complete under normal healing conditions [6, 7]. The continuous process of equilibrium between the synthesis of new stable collagen (collagen type I) and the lysis of old collagen (collagen type III) is characteristic of this phase, with the eventual formation of scar tissue [9].

Foetal and adult skin undergo different wound healing processes [10]. In contrast to wound repair in adult epithelia, there is an absence of scar formation in foetal tissues, which is characterized by rapid reepithelialization, lack of inflammation, and restoration of normal tissue architecture [11]. It is known that there is a significant difference in collagen deposition and its cross-linking patterns, hyaluronic acid content, extra cellular matrix (ECM) proteins, and adhesion proteins [11]. In comparison to foetal wound healing, adult and late-gestational skin heals less rapidly with a marked increase in inflammatory response and fibrosis resulting in the formation of a scar [12, 13]. While studies have been conducted to investigate the molecular differences between the adult and foetal wound healing processes, to date the mechanism of foetal scarless wound healing remains unclear [12].

Reports on foetal skin and scarless wound healing focus primarily on the skin's composition and the unique environment that the foetus is found in; to the best of our knowledge there are no studies reporting the differences in protein composition between foetal and adult fibroblasts. Fibroblasts play a critical role in the wound healing process through the release of signal molecules directing formation of collagen that closes the wound edges together [14]. Thus, clarifying differences in protein expression between adult and foetal fibroblasts will support the development of new therapeutic strategies to promote functional tissue regeneration during adult wound healing. In this study, we utilise two-dimensional gel electrophoresis (2DE) to determine differences in protein expression between human adult and foetal fibroblasts and identify proteins that may help unlock the secrets to scarless wound healing.

## 2. Methods and Materials

### 2.1. Materials and Reagents

Foetal bovine serum (FBS) and Penicillin/Streptococcus antibiotic were purchased from Gibco-Invitrogen (Sydney, Australia). Dulbecco's Modified Eagle's Medium (DMEM) was purchased from Lonza (Maryland, USA). Trypsin was acquired from Sigma-Aldrich (St. Louis, MO, USA). All other chemicals were of analytical grade and acquired from Univar (Sydney, Australia).

### 2.2. Fibroblasts Cultivation

Human adult dermal fibroblasts (HAF) were purchased from Lonza Australia Pty. Ltd (Sydney, Australia). Human foetal dermal fibroblasts (HFF) between 5th and 8th passages were obtained from the cell culture collection in the Faculty of Medicine at the University of New South Wales. All human foetal tissue experiments were performed with the approval of the UNSW Ethics Committee (EAC 08/284, The University of New South Wales, Australia). Both cell lines were separately cultured in fresh Dulbecco's modified Eagle's medium supplemented with 10% foetal bovine serum (DMEM-10% FBS) using 75 cm^2^ T-flasks with incubation (37°C, 5% CO_2_) until confluent. The cells were then washed twice with phosphate buffer solution (PBS) and cultured for 48 h in serum-free DMEM before harvest.

### 2.3. Protein Analysis: Sample Preparation

All sample preparations were conducted under clean conditions; cell samples (12 mL) were centrifuged (15 min, 30,000 g) and cell pellets were resuspended in PBS (10 mL) then centrifuged (2 min, 300 g) before resuspending in milliQ water (5 mL) and transferring to microcentrifuge tubes. To ensure complete removal of growth medium, resuspended cell pellets were centrifuged once more, supernatants discarded, and cell pellets resuspended in MilliQ water before a final centrifugation (18,000 g, 15 min). Supernatants were discarded and cell pellets were resolubilized in buffer containing 8 M urea, 100 mM DTT (dithiothreitol), 4% (w/v) CHAPS (3-[(3-Cholamidopropyl)-Dimethylammonio]-1-Propane Sulfonate), 0.2% (v/v) carrier ampholytes (pH 3–10; Bio-Rad, Hercules, CA), 40 mM Tris Base (pH 7), and 0.02% (w/v) bromophenol blue. Samples were then vortexed and sonicated (30 s) before protein separation through 2DE (XCell SureLock Novex minicell, Invitrogen).

### 2.4. Protein Analysis: 2D Electrophoresis

Isoelectric focusing was carried out by passive rehydrating of linear gradient Ready Strip IPG strips (18 cm, pH 3–10, BioRad) with 350 *μ*L of resolubilized protein samples of the same concentrations for 8 h. Rehydrated strips were loaded onto a horizontal electrophoresis unit (BioRad, California, USA) and focused for 100 kVh (kilovolt-hours) with cooling (15°C). Focused IPG strips were then equilibrated for 30 min with slow shaking in solution containing 6 M urea, 2% (w/v) SDS (sodium dodecyl sulphate), 0.375 M Tris-HCl (pH 8.8), 20% (v/v) glycerol, 100 mM DTT, and 2.5% (w/v) acrylamide. Equilibrated IPG strips were laid onto the second dimension (8–18% polyacrylamide gradient gels: 20 × 20 × 0.2). Proteins were separated in the second dimension by applying a current of 40 mA/gel for 2 h, followed by 50 mA/gel for a further 10 h at 15°C. For protein visualization, gels were stained with Coomassie G-250 dye (12 h) and destained in 1% (v/v) acetic acid before manual excision of visible protein spots. Gel pieces containing protein spots were transferred into 1.5 mL microcentrifuge tubes and stored at 4°C.

### 2.5. Spot Density Comparison

**Figure 1:**
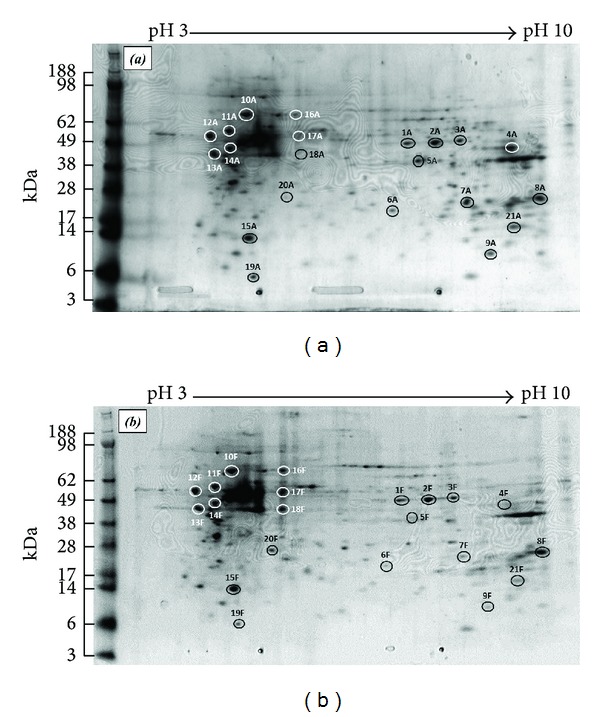
Representative 2DE gel maps of (a) human adult skin fibroblasts and (b) human foetal skin fibroblasts proteins using linear IPG 3–10 pH strips. Spot numbers correspond to identified proteins reported in Table 1.

The relative density of corresponding spots on both HAF and HFF 2DE were compared using the Image J 3D Surface Plot plug-in program, which expresses pixel intensity as height in three-dimensional plots (Figure 1) [15, 16]. This method was adapted from the NIH ImageJ website [15].

### 2.6. Protein Analysis: Mass Spectrometry

Excised gel pieces were first destained by washing twice in 120 *μ*L NH_4_HCO_3_ (ammonium bicarbonate, 25 mM) containing 50% (v/v) acetonitrile for 30 min, before drying in a Speedvac (Thermo Electron, Milford, MA). In-gel digestion of proteins was achieved by swelling gel pieces with 15 *μ*L of sequencing grade trypsin (20 ng/*μ*L, Promega, Annandale, NSW, Australia) in NH_4_HCO_3_ (25 mM, pH 7.8, 1 h, 37°C), followed by the addition of 20 *μ*L of NH_4_HCO_3_ (10 mM) and further incubation (18 h, 37°C). Gel pieces were then sonicated for 20 min to liberate peptide fragments and briefly centrifuged (15,000 g, 30 s).

The tryptic peptide digests were separated using HPLC (Ultimate 3000, Dionex; Amsterdam, Netherlands) equipped with a linear Ion Trap (LTQ FT Ultra, Thermo Electron; Bremen, Germany) mass spectrometer. Lists of peaks from the mass spectrometry data were developed using Mascot Daemon (Matrix Science, London, UK) and Extract msn (Thermo Scientific, Australia) programs with default parameters (where individual ion scores >30 indicated identity or extensive homology, *P* < 0.05). Lists were then analysed using the database search program Mascot (version 2.1, Matrix Science, London, UK). Search parameters included a precursor tolerance of 6 ppm, product ion tolerances of ±0.5 Da, oxidation of methionine residues specified as a variable modification, and an enzyme specificity of trypsin; in addition, one missed trypsin cleavage was permitted. The resulting data was analysed using the Swiss-Prot database (Swiss-Prot group, Switzerland). Known and predicted protein-protein interactions were established using the STRING search tool (v9.1, string-db.org).

### 2.7. Statistical Analysis

2DE was run in triplicate from the same protein preparation and the experiment was subsequently repeated (*n* = 2 × 3). Statistical analysis of spot densities was evaluated using the two-way ANOVA and Bonferroni posttest (significance level: 0.05).

## 3. Results and Discussion

The foetus has the ability to heal wounds by regenerating dermal layers with complete restoration of architecture, restoring the skin's original strength and function; in contrast, adult wounds heal with fibrosis and scars [16]. A number of studies have attempted to elucidate the molecular mechanisms of embryonic and adult wound healing to account for this difference [6, 10]. While protein maps of adult dermal and MRC5-fibroblasts, as well as data for mouse foetal fibroblasts, are available, a standardised comparison of proteins expressed by human adult and foetal dermal fibroblasts has yet to be reported [17, 18].

2DE, in association with computer-aided image analysis and mass spectrometric procedures for large-scale protein identification and characterization, permits the monitoring of a considerable percentage of the whole protein complement (proteome) within a biological system [19, 20]. In the study here, proteins secreted by adult and foetal fibroblasts were separated by 2DE (Figures 1(a) and 1(b), resp.). Gels were run in triplicate and the experiment was repeated; within each fibroblast lineage, gels showed no significant difference in protein spot patterns between gels and the repeat experiments, verifying the reproducibility of the system.

**Figure 2:**
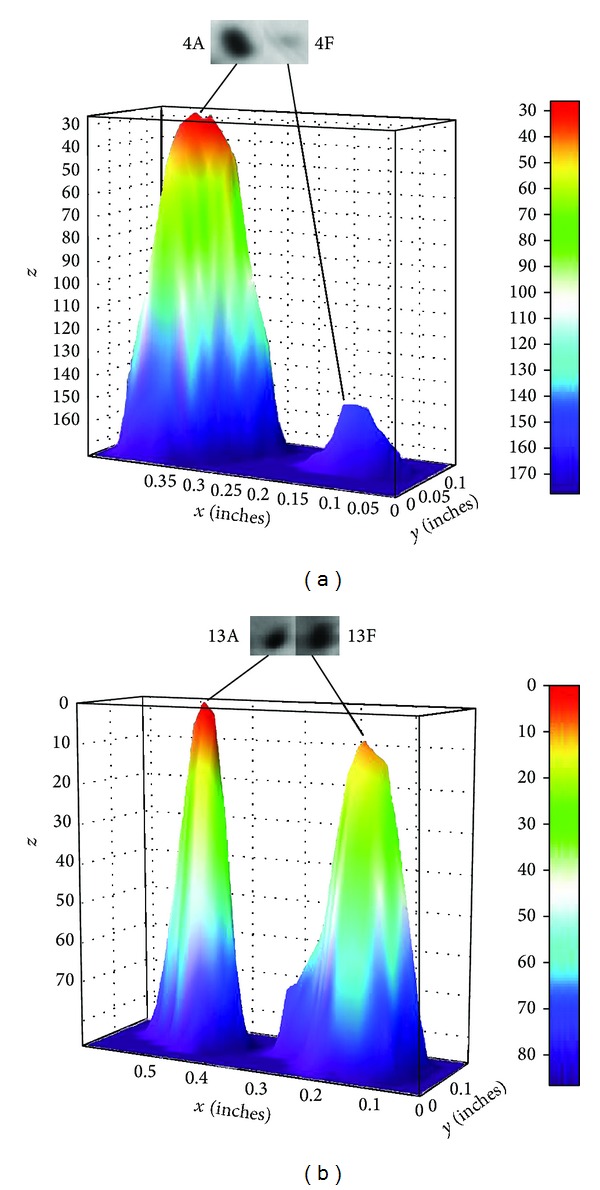
Image of 3D surface plots (inverted height) of corresponding gel spots 4 and 13 on HAF and HFF 2DE gels permitting quantitative analysis in respective protein concentrations.

While 2DE of the HAF and HFF proteomes showed similar profile patterns, variations in spot intensity were observed (Figure 1). For example, adult fibroblasts (HAF) produced a greater concentration of the protein identified as spot 4A in the gels than their foetal counterpart 4F. In contrast, differences in the densities of the protein identified as spots 13A and 13F cannot be resolved visually, (Figures 1(a) and 1(b), resp.). Application of the ImageJ 3D surface plot analysis system permitted quantitative evaluation of the relative protein spot densities between the two cell lines.  Figure 2 shows the 3D plots for the proteins represented as spots 4 and 13 on the 2DEs; these differences in intensity between adult and foetal protein expressions could subsequently be expressed as ratios as 1 : 0.11 ± 0.09 and 1 : 0.94 ± 0.08, respectively.

Based on a visual assessment of relative densities, 21 gel spots were excised from HAF and HFF proteome gels and their proteins subsequently identified (Table 1). Of the 21 gel spots analysed, 15 showed significant differences in their comparative protein concentrations between HAF and HFF (*P* < 0.05). Eleven of these proteins exhibited greater gel spot densities in adult fibroblasts compared to their foetal counterparts, with ratios ranging from 1 : 0.11 (3-ketoacyl-Co A thiolase) to 1 : 0.82 (Galectin-1, Table 1). Alpha-enolase was identified as the protein in 3 separate gel spots, 1–3; this was probably due to different posttranslational modifications [21]. Furthermore, 11 spots out of the 21 excised were proteins related to wound healing, and 3 of them (spots 7, 9, and 21) linked to scar formation.

The protein in gel spot 4 was identified as 3-Ketoacyl-CoA thiolase and was expressed in significantly greater concentrations by the adult fibroblast compared to their foetal counterparts (1 : 0.11 ± 0.09). This enzyme is located in the peroxisome or the mitochondria and catalyses the final reaction of fatty acid *β*-oxidation. [22]. Cao et al. have reported that the enzyme is a functional binding partner for BNIP3, known to mediate apoptosis and mitochondrial damage [23]. Similarly, fructose bisphosphate aldolase A (spot 5), Protein S100-A6 (spot 19), and glucosidase 2 subunit beta (spot 10) with HAF : HFF ratios of 1 : 0.68 ± 0.07, 1 : 0.78 ± 0.03, and 1 : 0.77 ± 0.07, respectively, were also more significantly expressed in the adult than in the foetal fibroblasts (*P* < 0.05; Table 1). Protein S100-A6 is a calcium binding protein that regulates various intracellular and extracellular activities such as protein phosphorylation and enzyme activity, the dynamics of cytoskeleton components, and cell proliferation and differentiation [24]. Glucosidase 2 subunit beta is a regulatory subunit of glucosidase 2, an endoplasmic reticulum (ER) heterodimeric enzyme that cleaves the two innermost *α*-1,3-linked glucose residues from N-linked oligosaccharides on nascent glycoproteins. This facilitates the binding and release of monoglucosylated glycoproteins with calnexin and Calreticulin [25]. The interaction of glucosidase 2 with Calreticulin, spot 12, suggests that the protein may exert an indirect influence on wound healing.

Tubulin alpha-1A chain (spot 17) was expressed in a significantly greater concentration in the foetal fibroblasts, such that the ratio HAF : HFF was 1 : 1.92 ± 0.04 (Table 1). Tubulin is a major constituent of microtubules and is important in a number of cellular processes. Microtubules are involved in maintaining cell structure and, together with microfilaments and intermediate filaments, form the cell cytoskeleton. Microtubules also provide platforms for intracellular transport and a variety of other cellular processes involving the movement of secretory vesicles and organelles, as well as the intracellular transport of metabolic products [26]. Similarly, heat shock cognate 71 kDa protein (HSP-71) (spot 16) and neuron cytoplasmic protein (spot 20) were also more prominent in the 2DE gels from foetal fibroblasts (HAF : HFF = 1 : 1.74 ± 0.06 and 1 : 1.51 ± 0.05, respectively; Table 1). Heat shock proteins are thought to play a crucial role in organism survival as they are ubiquitously present in cells under both normal and pathological conditions and their structure is evolutionarily conserved [27]. *In vivo* studies have shown that overexpression of HSPs may protect certain cells from selected noxious stimuli [28].

By definition in the literature, eight of the gel spots showing significant differences in protein expression between the adult and foetal fibroblasts were proteins related to wound healing [29–36]. Actin cytoplasmic-1 (spot 18) was expressed in significantly greater concentrations by the foetal fibroblasts compared to their adult counterparts (1 : 1.86 ± 0.11). Actins are highly conserved proteins ubiquitously expressed in all eukaryotic cells and are found as free monomers, “G-actin,” or as microfilaments “F-actin” [37]. Actins are essential for cellular functions such as motility, the maintenance of cell shape and polarity, cell division and cytokinesis, vesicle and organelle movement, cell signalling as well as the establishment and maintenance of cell junctions, and regulation of transcription [38]. Moreover, the interaction of F-actin with myosin forms the basis of muscle contraction and can also produce movement either by itself or with the help of molecular motors [38]. Therefore, actin plays an important role in embryogenesis as well as wound healing, where cell motility is crucial for sealing of wound margins [36]. Its relatively higher expression in foetal fibroblasts is consistent with its important role in foetal development.

Many proteins are known to regulate the polymerisation and depolymerisation of actin filaments [38]. Fructose bisphosphate aldolase A (spot 5) acts directly with actin filaments and therefore may function as a scaffolding protein [29]. Condeelis have reported that this protein binds to actin filaments in stress fibres within the cell and reversibly inhibited the contraction of fibroblast cells [39]. This suggests that fructose-bisphosphate aldolase A is a necessary structural component of the cytoskeleton and plays an important role in maintaining cytoskeletal structure as well as modulating cell mobility [29]. Its relatively lower concentration in foetal fibroblasts compared to adult cells supports this suggestion of a regulatory role (1 : 0.68 ± 0.07).

Cofilin-1 (spot 6) is another protein regulating actin and was found in a similar spot intensity ratio as fructose bisphosphate aldolase A (1 : 0.57 ± 0.07; Table 1). Cofilin-1 is a pH-sensitive, actin-depolymerizing protein that can bind to both G- and F-actins, and it is a regulator of stress fibre formation and collagen contraction [40]. Its activation is required for cell motility, which is a necessary step for tissue repair and regeneration [39]. Cofilin-1 is known to be a key regulator of actin dynamics at the leading edge of motile cells; it has a tightly regulated function of creating new actin barbed ends for polymerization and also depolymerizes old actin filaments [41]. It has also been shown that Cofilin-1 is able to amplify local actin polymerization responses upon cell stimulation, giving it the ability to set the direction of motility in crawling cells [41]. Phosphorylated and dephosphorylated cofilin-1 are also found in platelets, where the amount of the latter parallels the later stages of platelet aggregation in wound healing, suggesting that newly formed dephosphorylated cofilin-1 could have an important role in the cytoskeletal remodelling that occurs during platelet aggregation, a vital step in the early stages of wound healing [42].

Profilin-1 (spot 21) is an actin binding protein that affects cytoskeleton structure through regulation of actin polymerisation [43]. Profilin-1 promotes actin assembly via its ability to accelerate nucleotide exchange (ADP to ATP) on G-actin and shuttle Profilin-actin (ATP-bound) complex to free barbed ends of actin filaments [35]. Profilin-1 has also been implicated in the regulation of cell migration and proliferation during the wound healing process; studies have shown that deletion of the gene responsible for its expression impaired migration and proliferation in lower eukaryotic and endothelial cells, suggesting a role in angiogenesis [44]. Angiogenesis and fibrosis are two features of dermal wound repair reported in adult nonhuman primates but not found in scarless foetal repair [13]. Thus, the significantly lower concentration of profilin-1 expressed by the foetal fibroblasts compared to their adult counterparts in the study here supports this suggestion.

**Figure 3:**
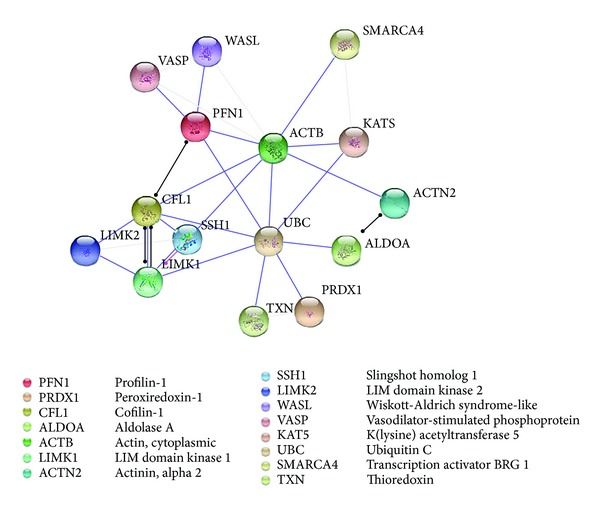
Protein association network in STRING analysis showing interactions of Actin, cytoplasmic (ACTB), Fructose-bisphosphate aldolase A (ALDOA), Cofilin-1 (CFL1), and Peroxiredoxin 1 (PRDX1) linked via Ubiquitin C (UBC). Line colours indicate the mode of action of interaction between proteins. 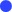 binding interaction, 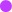 posttranslational modification, and 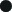 reactive interaction. Grey lines indicate interactions, but with low confidence scores.

The relationships between actin cytoplasmic-1 (ACTB), fructose bisphosphate aldolase A (ALDO A), cofilin-1 (CFL 1), and profilin-1 (PFN 1) are clearly illustrated through the STRING diagram in Figure 3. All the proteins identified have the ability to bind to ubiquitin c, with actin cytoplasmic-1 being able to bind directly with profilin-1 and cofilin-1. Ubiquitination is known to be a fundamental protein modification which regulates most cellular processes [45]. This comprehensive action was recently demonstrated by Kim et al. where their proteomic analysis of the human ubiquitin-modified proteome (ubiquitinome) identified about 19,000 sites in approximately 5000 proteins [46]. It has also been suggested that polyubiquitination can be considered as a sign of proteosomal or lysosomal protein degradation to control protein abundance [47, 48]. Therefore, the relative upregulation of the ubiquitin c binding proteins as observed in adult fibroblast here may imply some inhibition of ubiquitination, resulting in a delay in protein turnover.

**Figure 4:**
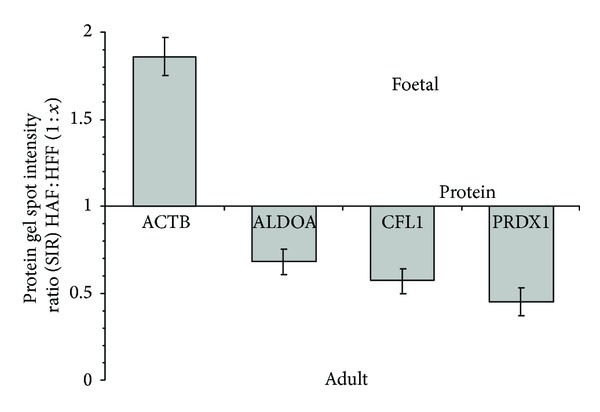
Variation in protein gel spot intensity ratio for wound healing proteins related as determined using STRING (Figure 3), ACTB: Actin cytoplasmic-1; ALDOA: Fructose-bisphosphate aldolase A; CFL 1: Cofilin-1; and PRDX 1: Peroxiredoxin-1.

The STRING diagram also shows a direct relationship between ubiquitin C and Peroxiredoxin-1 (PRDX-1; Figure 3). Similar to the other regulatory proteins, Peroxiredoxin-1 (spot 7) was found in relatively greater concentrations in adult fibroblasts with a spot intensity over double that derived from the foetal fibroblasts; HAF : HFF = 1 : 0.45 + 0.08. Peroxiredoxin-1 is an antioxidant enzyme catalysing the reduction of H_2_O_2_ and a wide range of organic peroxides [49]. Antioxidants govern intracellular redox statuses and in mammalian cells this has been linked to cellular differentiation, immune response, growth control, tumour promotion, and apoptosis [50, 51]. Peroxiredoxin-1 interacts with reducing agents that combat oxidative stress and free radicals; there is strong evidence of oxidative stress in impaired wound healing. However, low levels of reactive oxygen species (ROS) are still needed for effective defence against invading pathogens and mediation of intracellular signalling [14]. Furthermore, a number of studies report an increase in expression levels of Peroxiredoxin-1 in human primary cancers [52]. Thus, while Peroxiredoxin-1 has potent antioxidant abilities, its concentrations do not appear to vary during wound healing [31]. However, it is interesting to note that, in the study here, fructose bisphosphate aldolase 6, cofilin-1, Peroxiredoxin-1, and profilin-1 were all found in foetal fibroblasts at concentrations approximately half that found in adult cells, while in contrast, actin cytoplasmic-1 was found to be nearly double (Figure 4). Although not directly linked to ubiquitin C through STRING analysis, the expression of Lactotransferrin (spot 9) was also found to differ significantly between foetal and adult fibroblasts (HAF : HFF = 1 : 0.70 ± 0.08) and is reported to be expressed by a variety of glandular epithelial cells and skin epidermal keratinocytes [53].

Lactotransferrin can directly inhibit microbial adhesion and proliferation and is an important part of the mammalian immune system [54]. In the inflammatory phase of the wound healing process, Lactotransferrin also increases the production of proinflammatory cytokines, stimulates maturation of dendritic cells, and aids recruitment of various leukocytes [55]. However, Lactotransferrin also has the ability to neutralize an overactive immune response by preventing further release of cytokines that induce recruitment and activation of immune cells at the inflammatory sites [32]. In addition to its involvement in regulating the body's immune response to wounds, Takayama and Mizumachi suggest that Lactotransferrin also promotes fibroblast-mediated collagen contraction [56]. Contraction of collagen in wound healing is necessary to minimise the wound margins, permitting reepithelialization [57]. However, this contraction largely contributes to the formation of the bulk tissue that is observed as a scar after completion of the normal wound healing process. In contrast, Ferguson and Howarth have reported a minimal inflammatory response in scarless foetal wounds which correlated with a marked reduction in macrophage and monocyte infiltrates [58]. Thus, in the study here, the lower concentration of Lactotransferrin expressed by foetal fibroblasts relative to adult cells appears consistent with the promotion of scarless wound healing.

Gel spots 12 and 15 were identified as the wound healing proteins, Calreticulin and Galectin-1, respectively, and showed slight but significantly greater expression in the adult fibroblasts compared to foetal cells (Table 1). Galectin-1 is a potent regulator of cell adhesion, growth, and migration via protein/glycan and protein/protein interactions [59]. Galectin-1 has been detected in comparatively high concentrations in the stroma of squamous cell carcinomas and in healing wounds, with its activity increasing during scar formation [34]. Furthermore, Dvořánková et al. report the use of recombinant human Galectin-1 to significantly increase wound contraction in rats [59]. Calreticulin is a facilitator of phagocytosis in apoptotic cells; it facilitates the migration of phagocytes to the wound area upon its release from activated neutrophils. It is also released from cytotoxic lymphocytes upon association with target cells and subsequently enhances their ability to debride the wound [60]. Furthermore, Calreticulin also promotes accelerated wound closure and an increase in granulation tissue [33]. Both *in vitro* and *in vivo* studies have shown that Calreticulin has a positive effect on cell migration into wounds and production of extracellular matrix for tissue remodelling and supports even dispersion of collagen [33].

Gel spots 11, 13, and 14 were also found to contain proteins, whose functions are purported to be involved in wound healing. However, there appeared to be no significant differences in their expression between the foetal and adult fibroblasts (Table 1). Calumenin (spot 13) can modulate fibroblast activity by changing the intracellular levels of actin fragments as well as expression levels of the actin organizing protein septin 2 [61]. Therefore, calumenin is suggested to influence cytoskeleton rearrangement and cell proliferation during wound healing [61]. Gel spots 11 and 14 were identified as variants of protein disulphide isomerase (PDI), endoplasmic reticulum enzymes that catalyse intramolecular disulphide bonding reactions, such as the trimer formation of procollagen from polypeptides [62]. Protein disulphide isomerases have been reported to occur on the surface of blood platelets which suggests that proteins involved in haemostasis and wound healing are possible substrates for these enzymes [63]. Huang et al. report that protein disulphide isomerases purified from platelets catalysed the intramolecular disulphide exchange in thrombospondin-1 [64]. Thrombospondin-1 is an adhesive protein released from activated platelets where it forms disulphide linked complexes with thrombin and thrombin-serpin complexes [63]. These complexes constitute part of the fibrin clot that shield the wound as well as providing a provisional matrix for cells to migrate to the wounded area during the repair process, making protein disulphide isomerase an important player in the initial stages of wound healing [65]. Similarly, studies have also shown that fibronectin, a major component of extracellular matrix and part of the coagulation cascade during wound healing, has an intrinsic PDI activity [66].

## 4. Conclusions

In the last few decades, our comprehension of the basic biological processes involved in wound repair and tissue regeneration has expanded due to advances in cellular and molecular biology [62]. In contrast to wound repair in adult epithelia, there is an absence of scar formation in foetal tissues and better functional recovery, and while there are a number of studies reporting the molecular differences between the adult and foetal wound healing processes, to date the mechanism of foetal scarless wound healing remains unclear.

In the study here, standardised 2DE electrophoresis was used to compare the proteomes of human adult and foetal fibroblasts. While similar protein distributions were obtained, variations in the intensities of some protein spots between the adult and foetal maps were observed; the proteins in 21 of these were subsequently identified. Of the 21 spots selected, approximately half were found to play various roles in wound healing. While calumenin and protein disulphide-isomerase/A6 showed no significant differences in expression between the adult and foetal fibroblasts, 7 others showed significantly lower expression levels in the foetal cells, with ratios ranging from 1 : 0.45 ± 0.08 for Peroxiredoxin-1 to 1 : 0.82 ± 0.08 for Galectin-1. In contrast, actin cytoplasmic-1 was expressed by foetal cells in a comparatively greater concentration, 1 : 1.86 ± 0.11. Similarly, heat shock cognate protein, Tubulin alpha-1A chain, and neuron cytoplasmic protein, not categorised as wound healing proteins, were all expressed by foetal fibroblasts at significantly higher levels than their adult counterparts.

While it is debatable whether the culture conditions utilised here are representative of those experienced by tissues during epithelial injury and healing, it is clear that under the same conditions protein expression by foetal and adult fibroblasts differ in their expression levels. Furthermore, of those proteins showing observable differences many were related to wound healing and tissue regeneration. The comparative expression levels of these fibroblast proteins appear consistent with their known and suspected functions and the differences in wound healing behaviour between adult and foetal tissues.

While the principal aim in wound management is to achieve rapid wound closure with a functional tissue that has minimal aesthetic scarring, the ultimate goal in wound healing biology is to induce a more perfect reconstruction of the tissue in the wound area [67]. Thus, understanding the protein interactions that support scarless wound healing in foetal tissues, as well as the differences with their adult counterparts, will provide an important step in achieving this goal.

## Supplementary Material

Figure 1: Peptide sequence data from actin cytoplasmic 1 (ACT B) showing (A) a representation of the ACT B tryptic digest peptide sequence that were identified in highlighted red, and (B) an example of the ACT B peptide fragmentation spectra with lead ions shown as red lines. These represent the fragmentation spectra where (b) ions represent a fragmentation from the amine-terminus and (y) ions are a representation of fragmentation from the carboxyl-terminus of the protein [68].
Figure 2: Peptide sequence data from fructose bisphosphaste aldolase 1 (ALDO A) showing (A) a representation of the ALDO A tryptic digest peptide sequence that were identified in highlighted red, and (B) an example of the ALDO A peptide fragmentation spectra with lead ions shown as red lines. These represent the fragmentation spectra where (b) ions represent a fragmentation from the amine-terminus and (y) ions are a representation of fragmentation from the carboxyl-terminus of the protein [68].
Figure 3: Peptide sequence data from cofilin 1 (CFL 1) showing (A) a representation of the CFL 1 tryptic digest peptide sequence that were identified in highlighted red, and (B) an example of the CFL 1 peptide fragmentation spectra with lead ions shown as red lines. These represent the fragmentation spectra where (b) ions represent a fragmentation from the amine-terminus and (y) ions are a representation of fragmentation from the carboxyl-terminus of the protein [68].
Figure 4: Peptide sequence data from profilin 1 (PFN 1) showing (A) a representation of the PFN 1 tryptic digest peptide sequence that were identified in highlighted red, and (B) an example of the PFN 1 peptide fragmentation spectra with lead ions shown as red lines. These represent the fragmentation spectra where (b) ions represent a fragmentation from the amine-terminus and (y) ions are a representation of fragmentation from the carboxyl-terminus of the protein [68].

## Figures and Tables

**Table 1:** Variation in relative protein concentrations expressed by human adult (HAF) and foetal (HFF) dermal fibroblasts, as identified by mass spectrometry.

Spot number	Protein	Accession number	*pI *	*pI *	Mr	Mr	SIR	±
1	Alpha-enolase	P06733	7.01	7.10	47169	49000	1 : 0.86	0.12
2	Alpha-enolase	P06733	7.01	7.61	47169	49000	1 : 0.93	0.09
3	Alpha-enolase	P06733	7.01	8.00	47169	49000	1 : 0.84	0.11
4	3-ketoacyl-CoA thiolase, mitochondrial	P42765	8.32	9.00	41924	47000	1 : 0.11	0.09*
5	Fructose-bisphosphate aldolase A^●^	P04075	8.30	7.34	39420	40000	1 : 0.68	0.07*
6	Cofilin-1^●^	P23528	8.22	6.85	18502	20000	1 : 0.57	0.07*
7	Peroxiredoxin-1^●^	Q06830	8.27	8.17	22110	23000	1 : 0.45	0.08*
8	Peptidyl-prolyl cis-trans isomerase B	P23284	9.60	9.50	22068	24000	1 : 0.83	0.10*
9	Lactotransferrin^●^	P02788	8.50	8.67	78182	10000	1 : 0.71	0.05*
10	Glucosidase 2 subunit beta	P14314	4.33	4.34	59425	65000	1 : 0.77	0.07*
11	Protein disulfide-isomerase^●^	P07237	4.76	4.00	57116	57000	1 : 0.98	0.03
12	Calreticulin^●^	P27797	4.29	3.68	48142	53000	1 : 0.81	0.09*
13	Calumenin^●^	O43852	4.47	3.70	37107	45000	1 : 0.92	0.11
14	Protein disulfide-isomerase A6^●^	Q15084	4.95	4.00	48121	47000	1 : 0.94	0.08
15	Galectin-1^●^	P09382	5.33	4.34	14716	14000	1 : 0.82	0.08*
16	Heat shock cognate 71 kDa protein	P11142	5.37	5.17	70898	65000	1 : 1.74	0.06*
17	Tubulin alpha-1A chain	Q71U36	4.94	5.17	50136	53000	1 : 1.92	0.04*
18	Actin cytoplasmic-1^●^	P60709	5.29	5.17	41737	45000	1 : 1.86	0.11*
19	Protein S100-A6	P06703	5.32	4.40	10180	6000	1 : 0.78	0.03*
20	Neuron cytoplasmic protein 9.5	P09936	5.33	5.00	24824	26000	1 : 1.51	0.05*
21	Profilin-1^●^	P07737	8.44	9.10	15054	15000	1 : 0.70	0.10*

^●^Proteins with functions related to wound healing; SIR: spot intensity ratio HAF : HFF; *statistical significance between relative spot intensities (*n* = 2 × 3, *P* > 0.005).

## References

[1] Sund B (2000). *New Developments in Wound Care*.

[2] Reichert-Penetrat S, Barbaud A, Martin S, Omhover L, Weber M, Schmutz J-L (1998). Pemphigus vulgaris on an old surgical scar: Koebner’s phenomenon?. *European Journal of Dermatology*.

[3] Mockenhaupt M, Viboud C, Dunant A (2008). Stevens-Johnson syndrome and toxic epidermal necrolysis: assessment of medication risks with emphasis on recently marketed drugs. The EuroSCAR-study. *Journal of Investigative Dermatology*.

[4] Kaneki T, Kawashima A, Hayano T (1998). Churg-Strauss syndrome (allergic granulomatous angitis) presenting with ileus caused by ischemic ileal ulcer. *Journal of Gastroenterology*.

[5] Lad R (2006). Biotechnology in skin care (I): overview. *Biotechnology in Personal Care*.

[6] Martin P (1997). Wound healing—aiming for perfect skin regeneration. *Science*.

[7] Gurtner GC, Werner S, Barrandon Y, Longaker MT (2008). Wound repair and regeneration. *Nature*.

[8] Cumming BD, McElwain DLS, Upton Z (2009). A mathematical model of wound healing and subsequent scarring. *Journal of the Royal Society Interface*.

[9] Rovee T, Press H, Shai A, Maibach HI (2005). Natural course of wound repair versus impaired healing in chronic skin ulcers. *Wound Healing and Ulcers of the Skin*.

[10] Ferguson MWJ, Whitby DJ, Shah M, Armstrong J, Siebert JW, Longaker MT (1996). Scar formation: the spectral nature of fetal and adult wound repair. *Plastic and Reconstructive Surgery*.

[11] Colwell AS, Longaker MT, Lorenz HP (2003). Fetal wound healing. *Frontiers in Bioscience*.

[12] Draaijers LJ, Tempelman FRH, Botman YAM (2004). The patient and observer scar assessment scale: a reliable and feasible tool for scar evaluation. *Plastic and Reconstructive Surgery*.

[13] Lorenz HP, Whitby DJ, Longaker MT, Adzick NS (1993). Fetal wound healing: the ontogeny of scar formation in the non-human primate. *Annals of Surgery*.

[14] Clark R (1996). Wound repair: overview and general considerations. *The Molecular and Cellular Biology of Wound Repair*.

[15] National Institute of Health (2013). *Surface Plot*.

[16] Honardoust D, Tredget EE, Sen CK (2011). Adult skin wounds can be induced to regenerate through modulation of cells and extracellular matrix molecules. *Advances in Wound Care*.

[17] Boraldi F, Bini L, Liberatori S (2003). Proteome analysis of dermal fibroblasts cultured in vitro from human healthy subjects of different ages. *Proteomics*.

[18] Benvenuti S, Cramer R, Quinn CC (2002). Differential proteome analysis of replicative senescence in rat embryo fibroblasts. *Molecular & Cellular Proteomics*.

[19] Dutt MJ, Lee KH (2000). Proteomic analysis. *Current Opinion in Biotechnology*.

[20] Honoré B (2001). Genome- and proteome-based technologies: status and applications in the postgenomic era. *Expert Review of Molecular Diagnostics*.

[21] Mann M, Jensen ON (2003). Proteomic analysis of post-translational modifications. *Nature Biotechnology*.

[22] Thompson S, Mayerl F, Peoples OP, Masamune S, Sinskey AJ, Walsh CT (1989). Mechanistic studies on *β*-ketoacyl thiolase from Zoogloea ramigera: identification of the active-site nucleophile as CyS89, its mutation to Ser89, and kinetic and thermodynamic characterization of wild-type and mutant enzymes. *Biochemistry*.

[23] Cao W, Liu N, Tang S (2008). Acetyl-coenzyme A acyltransferase 2 attenuates the apoptotic effects of BNIP3 in two human cell lines. *Biochimica et Biophysica Acta*.

[24] Donato R (2001). S100: a multigenic family of calcium-modulated proteins of the EF-hand type with intracellular and extracellular functional roles. *The International Journal of Biochemistry and Cell Biology*.

[25] Pelletier MF, Marcil A, Sevigny G (2000). The heterodimeric structure of glucosidase II is required for its activity, solubility, and localization in vivo. *Glycobiology*.

[26] Alieva IB, Zemskov EA, Kireev II (2010). Microtubules growth rate alteration in human endothelial cells. *Journal of Biomedicine and Biotechnology*.

[27] Kiang JG, Tsokos GC (1998). Heat shock protein 70 kDa: molecular biology, biochemistry, and physiology. *Pharmacology and Therapeutics*.

[28] Ciocca DR, Green S, Elledge RM (1998). Heat shock proteins hsp27 and hsp70: lack of correlation with response to tamoxifen and clinical course of disease in estrogen receptor-positive metastatic breast cancer (a southwest oncology group study). *Clinical Cancer Research*.

[29] Kusakabe T, Motoki K, Hori K (1997). Mode of interactions of human aldolase isozymes with cytoskeletons. *Archives of Biochemistry and Biophysics*.

[30] Nishida E, Maekawa S, Sakai H (1984). Cofilin, a protein in porcine brain that binds to actin filaments and inhibits their interactions with myosin and tropomyosin. *Biochemistry*.

[31] Kümin A, Schäfer M, Epp N (2007). Peroxiredoxin 6 is required for blood vessel integrity in wounded skin. *Journal of Cell Biology*.

[32] Legrand D, Elass E, Pierce A, Mazurier J (2004). Lactoferrin and host defence: an overview of its immuno-modulating and anti-inflammatory properties. *Biometals*.

[33] Gold LI, Rahman M, Blechman KM (2006). Overview of the role for calreticulin in the enhancement of wound healing through multiple biological effects. *Journal of Investigative Dermatology Symposium Proceedings*.

[34] Klíma J, Lacina L, Dvořánková B (2009). Differential regulation of galectin expression/reactivity during wound healing in porcine skin and in cultures of epidermal cells with functional impact on migration. *Physiological Research*.

[35] Witke W (2004). The role of profilin complexes in cell motility and other cellular processes. *Trends in Cell Biology*.

[36] Alberts B, Johnson A, Lewis J, Raff M, Roberts K, Walter P (2002). The cytoskeleton and cell behavior. *Molecular Biology of the Cell*.

[37] Zhu L, Zhang Y, Hu Y, Wen T, Wang Q (2013). Dynamic actin gene family evolution in primates. *BioMed Research International*.

[38] Dominguez R, Holmes KC (2011). Actin structure and function. *Annual Review of Biophysics*.

[39] Condeelis J (2001). How is actin polymerization nucleated in vivo?. *Trends in Cell Biology*.

[40] Bosselut N, Housset C, Marcelo P (2010). Distinct proteomic features of two fibrogenic liver cell populations: hepatic stellate cells and portal myofibroblasts. *Proteomics*.

[41] Danen EHJ, van Rheenen J, Franken W (2005). Integrins control motile strategy through a Rho-cofilin pathway. *Journal of Cell Biology*.

[42] Davidson MML, Haslam RJ (1994). Dephosphorylation of cofilin in stimulated platelets: roles for a GTP-binding protein and Ca^2+^. *Biochemical Journal*.

[43] Goldschmidt-Clermont PJ, Machesky LM, Baldassare JJ, Pollard TD (1990). The actin-binding protein profilin binds to PIP2 and inhibits its hydrolysis by phospholipase C. *Science*.

[44] Ding Z, Lambrechts A, Parepally M, Roy P (2006). Silencing profilin-1 inhibits endothelial cell proliferation, migration and cord morphogenesis. *Journal of Cell Science*.

[45] Pickart CM (2001). Mechanisms underlying ubiquitination. *Annual Review of Biochemistry*.

[46] Kim W, Bennett EJ, Huttlin EL (2011). Systematic and quantitative assessment of the ubiquitin-modified proteome. *Molecular Cell*.

[47] Xu P, Duong DM, Seyfried NT (2009). Quantitative proteomics reveals the function of unconventional ubiquitin chains in proteasomal degradation. *Cell*.

[48] Segré CV, Chiocca S (2011). Regulating the regulators: the post-translational code of class I HDAC1 and HDAC2. *Journal of Biomedicine and Biotechnology*.

[49] Wood ZA, Schröder E, Harris JR, Poole LB (2003). Structure, mechanism and regulation of peroxiredoxins. *Trends in Biochemical Sciences*.

[50] Kalebic T, Kinter A, Poli G, Anderson ME, Meister A, Fauci AS (1991). Suppression of human immunodeficiency virus expression in chronically infected monocytic cells by glutathione, glutathione ester, and N-acetylcysteine. *Proceedings of the National Academy of Sciences of the United States of America*.

[51] Wang Y, Manevich Y, Feinstein SI, Fisher AB (2004). Adenovirus-mediated transfer of the 1-cys peroxiredoxin gene to mouse lung protects against hyperoxic injury. *The American Journal of Physiology—Lung Cellular and Molecular Physiology*.

[52] Kim J-H, Bogner PN, Baek S-H (2008). Up-regulation of peroxiredoxin 1 in lung cancer and its implication as a prognostic and therapeutic target. *Clinical Cancer Research*.

[53] Cumberbatch M, Dearman RJ, Uribe-Luna S (2000). Regulation of epidermal Langerhans cell migration by lactoferrin. *Immunology*.

[54] Legrand D, Mazurier J (2010). A critical review of the roles of host lactoferrin in immunity. *Biometals*.

[55] de la Rosa G, Yang D, Tewary P, Varadhachary A, Oppenheim JJ (2008). Lactoferrin acts as an alarmin to promote the recruitment and activation of APCs and antigen-specific immune responses. *The Journal of Immunology*.

[56] Takayama Y, Mizumachi K (2001). Effects of lactoferrin on collagen gel contractile activity and myosin light chain phosphorylation in human fibroblasts. *FEBS Letters*.

[57] Beare AHM, O’Kane S, Krane SM, Ferguson MWJ (2003). Severely impaired wound healing in the collagenase-resistant mouse. *Journal of Investigative Dermatology*.

[58] Ferguson M, Howarth G (1992). Marsupial models of scarless fetal wound healing. *Fetal Wound Healing*.

[59] Dvořánková B, Szabo P, Lacina L (2011). Human galectins induce conversion of dermal fibroblasts into myofibroblasts and production of extracellular matrix: potential application in tissue engineering and wound repair. *Cells Tissues Organs*.

[60] Gardai SJ, McPhillips KA, Frasch SC (2005). Cell-surface calreticulin initiates clearance of viable or apoptotic cells through trans-activation of LRP on the phagocyte. *Cell*.

[61] Coppinger JA, Cagney G, Toomey S (2004). Characterization of the proteins released from activated platelets leads to localization of novel platelet proteins in human atherosclerotic lesions. *Blood*.

[62] Clark R (1996). *The Molecular and Cellular Biology of Wound Repair*.

[63] Milev Y, Essex DW (1999). Protein disulfide isomerase catalyzes the formation of disulfide-linked complexes of thrombospondin-1 with thrombin-antithrombin III. *Archives of Biochemistry and Biophysics*.

[64] Huang EM, Detwiler TC, Milev Y, Essex DW (1997). Thiol-disulfide isomerization in thrombospondin: effects of conformation and protein disulfide isomerase. *Blood*.

[65] Detwiler TC, Chang AC, Speziale MV, Browne PC, Miller JJ, Chen K (1992). Complexes of thrombin with proteins secreted by activated platelets. *Seminars in Thrombosis and Hemostasis*.

[66] Langenbach KJ, Sottile J (1999). Identification of protein-disulfide isomerase activity in fibronectin. *The Journal of Biological Chemistry*.

[67] Tonnesen MG, Feng X, Clark RAF (2000). Angiogenesis in wound healing. *Journal of Investigative Dermatology Symposium Proceedings*.

