# Supplementary material for: Towards Scarless Wound Healing: A Comparison of Protein Expression between Human, Adult and Foetal Fibroblasts
Source: Biomed Res Int. 2014 Jan 30;2014:676493. doi: 10.1155/2014/676493 (PMC3925539; doi:10.1155/2014/676493)
Supplement: Supplementary file 1 — Figure 1: Peptide sequence data from actin cytoplasmic 1 (ACT B) showing (A) a representation of the ACT B tryptic digest peptide sequence that were identified in highlighted red, and (B) an example of the ACT B peptide fragmentation spectra with lead ions shown as red lines. These represent the fragmentation spectra where (b) ions represent a fragmentation from the amine-terminus and (y) ions are a representation of fragmentation from the carboxyl-terminus of the protein [68]. Figure 2: Peptide sequence data from fructose bisphosphaste aldolase 1 (ALDO A) showing (A) a representation of the ALDO A tryptic digest peptide sequence that were identified in highlighted red, and (B) an example of the ALDO A peptide fragmentation spectra with lead ions shown as red lines. These represent the fragmentation spectra where (b) ions represent a fragmentation from the amine-terminus and (y) ions are a representation of fragmentation from the carboxyl-terminus of the protein [68]. Figure 3: Peptide sequence data from cofilin 1 (CFL 1) showing (A) a representation of the CFL 1 tryptic digest peptide sequence that were identified in highlighted red, and (B) an example of the CFL 1 peptide fragmentation spectra with lead ions shown as red lines. These represent the fragmentation spectra where (b) ions represent a fragmentation from the amine-terminus and (y) ions are a representation of fragmentation from the carboxyl-terminus of the protein [68]. Figure 4: Peptide sequence data from profilin 1 (PFN 1) showing (A) a representation of the PFN 1 tryptic digest peptide sequence that were identified in highlighted red, and (B) an example of the PFN 1 peptide fragmentation spectra with lead ions shown as red lines. These represent the fragmentation spectra where (b) ions represent a fragmentation from the amine-terminus and (y) ions are a representation of fragmentation from the carboxyl-terminus of the protein [68]. [file 676493.f1.pdf]

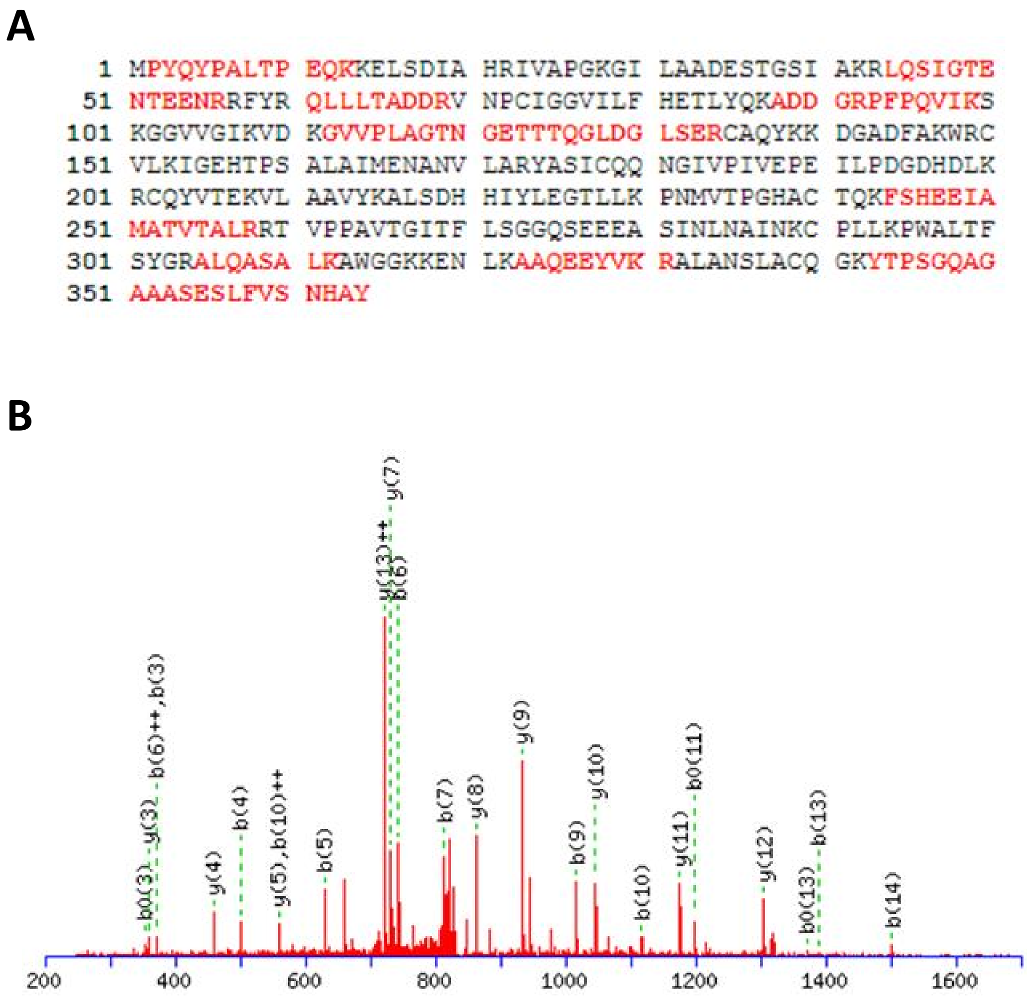

Figure 1: Peptide sequence data from actin cytoplasmic 1 (ACT B) showing (A) a representation of the ACT B tryptic digest peptide sequence that were identified in highlighted red, and (B) an example of the ACT B peptide fragmentation spectra with lead ions shown as red lines. These represent the fragmentation spectra where (b) ions represent a fragmentation from the amine-terminus and (y) ions are a representation of fragmentation from the carboxyl-terminus of the protein [68].

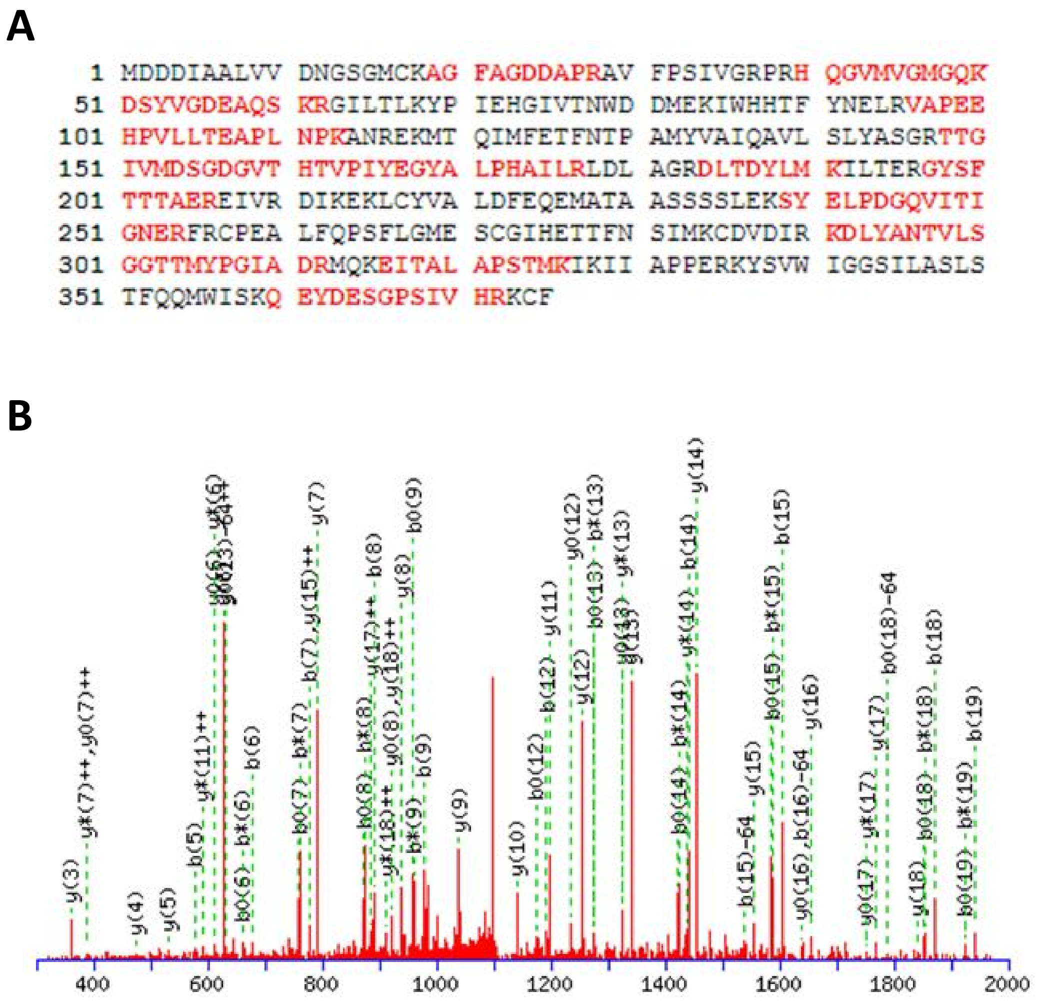

Figure 2: Peptide sequence data from fructose bisphosphaste aldolase 1 (ALDO A) showing (A) a representation of the ALDO A tryptic digest peptide sequence that were identified in highlighted red, and (B) an example of the ALDO A peptide fragmentation spectra with lead ions shown as red lines. These represent the fragmentation spectra where (b) ions represent a fragmentation from the amine-terminus and (y) ions are a representation of fragmentation from the carboxyl-terminus of the protein [68].

A

```
  1 MASGVAVSDG VIKVFNDMKV RKSSTPEEVK KRKKAVLFCL SEDKKNIILE
 51 EGKEILVGDV GQTVDDPYAT FVKMLPDKDC RYALYDATYE TKESKKEDLV
101 FIFWAPESAP LKSKMIYASS KDAIKKKLTG IKHELQANCY EEVKDRCTLA
151 EKLGGSAVIS LEGKPL
```

B

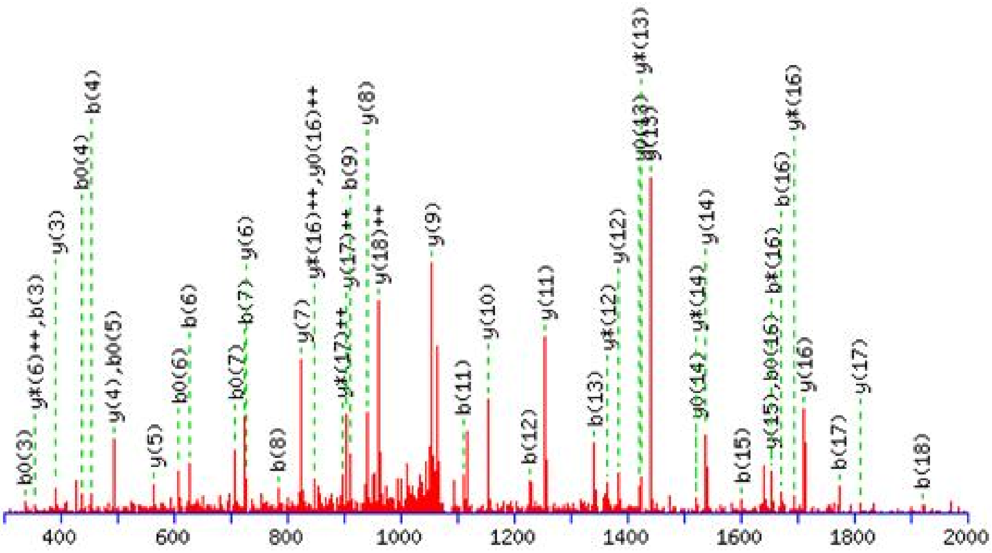

<u>Figure 3:</u> Peptide sequence data from cofilin 1 (CFL 1) showing (A) a representation of the CFL 1 tryptic digest peptide sequence that were identified in highlighted red, and (B) an example of the CFL 1 peptide fragmentation spectra with lead ions shown as red lines. These represent the fragmentation spectra where (b) ions represent a fragmentation from the amine-terminus and (y) ions are a representation of fragmentation from the carboxyl-terminus of the protein [68].

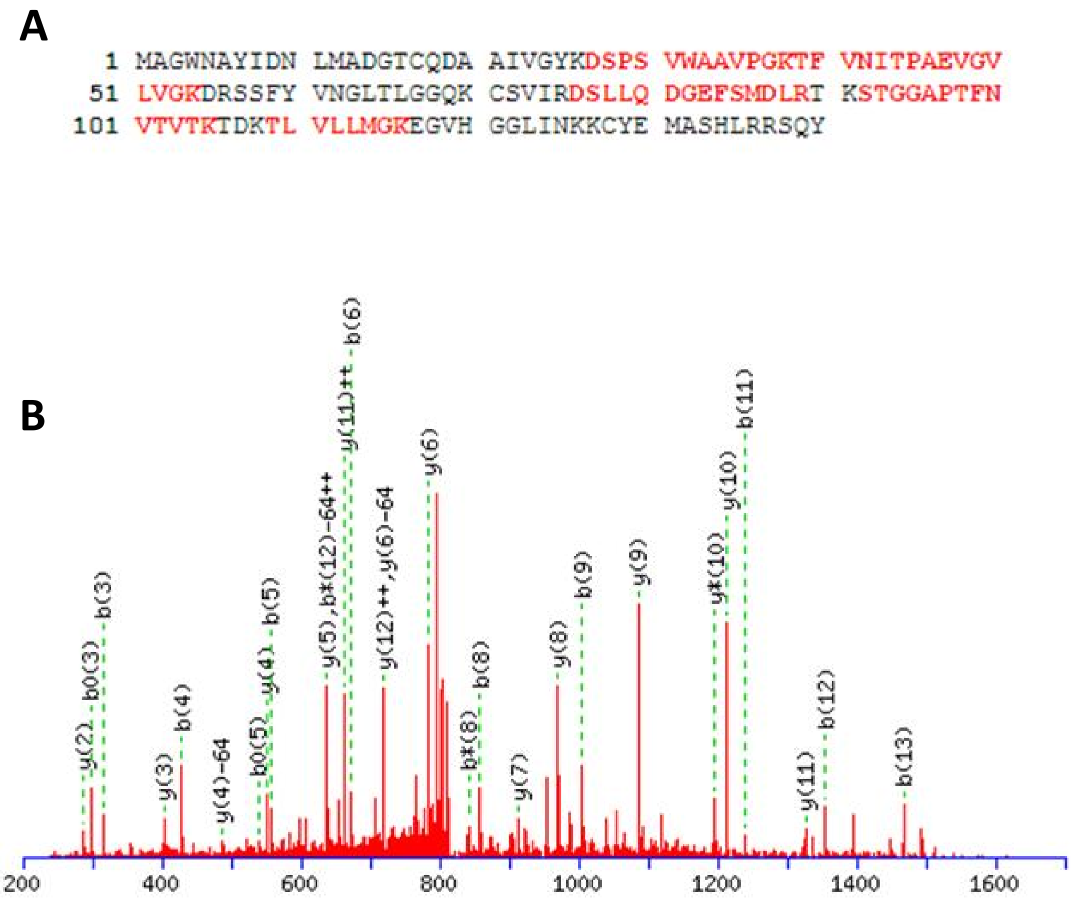

Figure 4: Peptide sequence data from profilin 1 (PFN 1) showing (A) a representation of the PFN 1 tryptic digest peptide sequence that were identified in highlighted red, and (B) an example of the PFN 1 peptide fragmentation spectra with lead ions shown as red lines. These represent the fragmentation spectra where (b) ions represent a fragmentation from the amine-terminus and (y) ions are a representation of fragmentation from the carboxyl-terminus of the protein [68].
